# Structural and functional insights of NINJ1 in plasma membrane rupture during cell death

**DOI:** 10.1186/s43556-023-00169-5

**Published:** 2024-03-01

**Authors:** Chehao Lee, Yuqing Liang, Yang Li

**Affiliations:** 1 Department of Traditional Chinese Medicine, Key Laboratory of Birth Defects and Related Diseases of Women and Children of Ministry of Education, West China Second University Hospital, Sichuan University, No. 1416, Section 1, Chenglong Avenue, Chengdu, 610041 Sichuan Province P. R. China; 2 School of Basic Medical Sciences, Chengdu University of Traditional Chinese Medicine, Chengdu, 611137 China; 3 Department of Respiratory, Chengdu Qingyang Hospital of Traditional Chinese Medicine, Chengdu, 610072 China

A recent study in *Nature* has revealed the Structure basis of NINJ1 in cell membrane rupture during cell death [1]. This was achieved using techniques like cryo-electron microscopy and molecular dynamics simulations. The research has deepened our understanding of the molecular processes that dictate programmed cell death.

Ninjurin1(NINJ1) was initially identified due to its unique response to spinal cord injuries in rats. Its involvement in neuroregeneration led to further studies which showed that NINJ1 is present in various tissues including the heart, liver, lungs, and kidneys. Structurally, NINJ1 is composed of distinct regions with the central part showing high conservation across species. The protein plays multiple roles, ranging from neuronal regeneration and cell adhesion to apoptosis [2]. Research indicates that NINJ1 is vital in the process of plasma membrane rupture (PMR) [3]. The absence of NINJ1 can delay PMR, thereby suppressing the spread of inflammation and preventing certain types of cell deaths. While the link between NINJ1 and PMR is clear, the exact mechanisms remain a topic of ongoing research.

Firstly, Degen et al. and his team used mouse bone-marrow-derived macrophages (BMDM) demonstrated that after inflammasome activation, NINJ1 forms dimers and trimers, which eventually evolve into larger polymers. This polymerization process correlates with Gasdermin D (GSDMD) pore formation. Utilizing fluorescence microscopy in Hela cell, the behavior of NINJ1 during these processes was observed in detail. To delve deeper, author used time-lapse fluorescence microscopy in HeLa cells, studying NINJ1 polymerization when co-expressed with GFP-tagged NINJ1 (hNINJ1) and a CRY2–caspase-1 fusion (opto-casp1). This approach induced GSDMD-mediated pyroptosis in individual cells through optogenetics. As the dye DRAQ7 penetrated the cells, indicating plasma membrane damage, NINJ1 began clustering at the membrane, maintaining this position even after cell breakage. This studies further confirmed the unique behavior of NINJ1 during pyroptosis. It's observed that NINJ1 starts clustering at the cell membrane, a behavior not seen in other proteins. Such behavior suggests an intrinsic mechanism of NINJ1, which, when activated, can lead to membrane rupture.

Cryo-electron microscopy (Cryo-EM) capture a clear structure of hNINJ1 filaments. This structure revealed linear stacks of subunits. It could be distinctly identified as four α-helices, α1-4 (Fig. 1a). Interestingly, the observed density showed two parallel hNINJ1 filaments. The filament's design was based on protomers stacking like a fence. Core helices α3 and α4 form the filament's backbone. Notably, these are hydrophobic helices. The N-terminal helices, α1 and α2, have a pronounced kink at L56, leading to a unique configuration with α1 connecting to adjacent protomers, creating a robust polymerization interface. Key intermolecular interactions were observed, with the K45-D53 salt bridge being significant. Additionally, α2's amphipathic helix formed hydrophobic interactions with α3. The contact between α3 and α4 in adjacent protomers was also identified. Interestingly, while the experimental NINJ1 structure coincided with the AlphaFold model for helices α2, α3, and α4, it deviated for α1. Molecular dynamics simulations indicated potential restructuring of the α1 helix towards the cryo-EM structure. Co-evolution analysis of NINJ1 underscored the filamentous structure's biological significance, particularly emphasizing the intermolecular connection between helices α3 and α4.

**Fig. 1:**
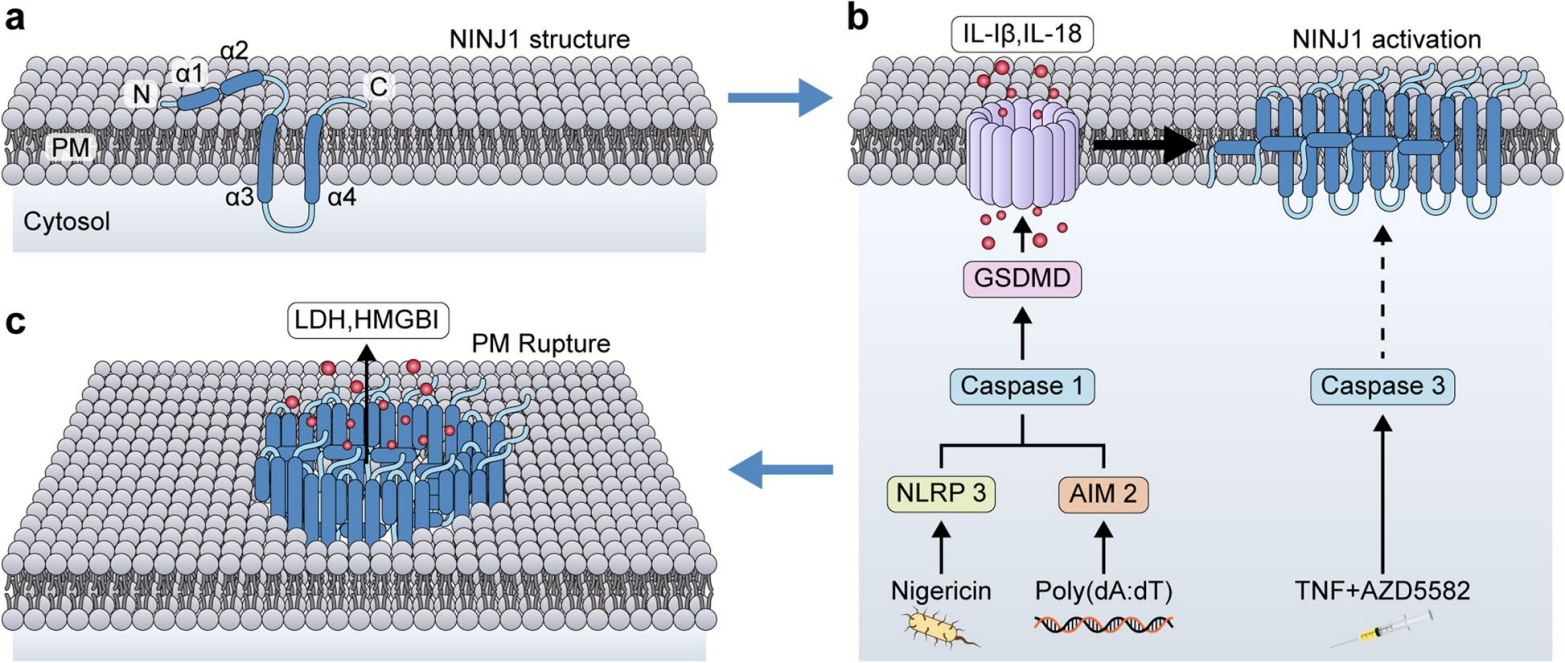
The model of PMR induced by NINJ1. **a**. Structural representation of NINJ1. **b**. Mechanism diagram illustrating NINJ1 oligomerization. **c**. NINJ1 oligomerization triggers PMR

Upon evaluating NINJ1 filament's surface hydrophobicity, it became evident that one side is hydrophilic while the opposite is hydrophobic. This dichotomy clarifies how two hNINJ1 filaments can stack hydrophobically, as observed in the cryo-EM results. Such a topology, featuring distinct hydrophilic and hydrophobic sides, is characteristic of pore-forming proteins. This suggests that NINJ1 filaments may cap membrane edges, facilitating lipid bilayer rupture. Supporting this, the hydrophobic curve's height approximately equals a typical eukaryotic plasma membrane's thickness. To test NINJ1's capability in influencing membrane permeability, NINJ1 was reconstituted into proteoliposomes containing specific lipids and a fluorescent lipid marker, nitrobenzoxadiazole-phosphatodylcholine (NBD-PC). When exposed to dithionite, which quenches NBD-PC fluorescence, an increase in NINJ1 proteoliposome permeability was observed with rising protein: lipid ratio. Further, molecular dynamics simulations were used to understand hNINJ1's behavior at membrane edges. Linear filaments remained intact and stable when positioned on opposing membrane edges. The simulation also modeled a small NINJ1-induced membrane pore by rearranging filaments into a ring, which exhibited structural stability. However, simulations with a truncated NINJ1 version, missing helices α1 and α2, saw a collapse and closure of the ring polymers, emphasizing the crucial role of these helices.Overall, the evidence suggests that NINJ1 filaments might effectively cap membrane edges in different configurations. Specifically, helix α1 not only provides stability to NINJ1 filaments but also offers adaptability, potentially essential for penetrating compact cellular membranes.

To validate the structural model, 14 mutants of hNINJ1 were tested for filament formation and their effects on cell lysis in vitro and in cells. Eight mutants (K44Q, K45Q, A47L, D53A, G95L, T123L, I134F and A138L) were designed to possibly disrupt the filament structure and two mutants at intramolecular interfaces (I84F and Q91A) were designed to potentially break the filament structure, while four (V82F, V82W, L121F and L121W) aimed to maintain NINJ1 polymerization. A majority of mutants, designed to hinder filament formation, showed reduced cell lysis and filament formation. However, A47L displayed discrepancies between in vitro and cellular results, suggesting cell-specific interactions. Four mutations maintaining hydrophobicity functioned as expected in vitro, but showed varied cell lysis levels, aligning with the structural model. In a physiological setting, the impact of these mutations on inflammasome-induced pyroptosis was tested by introducing them into primary NINJ1^−/−^ mouse BMDMs and activating the NACHT, LRR and PYD domains-containing protein 3 (NLRP3) inflammasome. Results were consistent with previous studies, except for the A47L mutant which might have species-specific variations. These findings solidified the agreement between in vitro and cell-based mutation results with the cryo-EM structure of NINJ1 filaments. Further testing on endogenous NINJ1 for potential dominant-negative effects showed some mutants might interfere with hNINJ1 filament functionality. Some mutants displayed unexpected PMR activity, suggesting these mutations could slightly weaken the intermolecular interactions, but still work within a mixed filament of wild-type and mutant proteins. In conclusion, the mutagenesis study confirmed that the NINJ1 filament formation in vitro is consistent with their ability to cause PMR in human and mouse cells.

In conclusion, under normal circumstances, NINJ1 remains inactive and monomeric within the cellular membrane (Fig. 1a). However, during cell death, NINJ1 polymerizes to form larger structures that promote membrane rupture and enable the release of cellular contents. (Fig. 1b-c). Interestingly, the nature of this transition from inactive to active remains elusive, but this work suggests that alterations in membrane composition might provide the activation cue. Furthermore, on the same day, *Nature* published another article related to NINJ1, which showed that inhibiting NINJ1 and PMR can reduce cell death, inflammation, and liver cell damage in mice [4]. The interaction mechanisms and upstream pathways of NINJ1 with other genes remain unclear, apart from the known role of GSDMD and Cellular tumor antigen p53 (TP53) in reducing cell death [1, 5]. However, previous studies have shown NINJ1 expression on the Golgi apparatus through immunofluorescence imaging [1]. Our previous research also detected NINJ1 expression surrounding both the cell membrane and the nucleus, exhibiting morphological similarities to either the endoplasmic reticulum or the Golgi apparatus [5]. This observation implies that NINJ1, synthesized within the nucleus and stimulated by external factors, may be secreted via the endoplasmic reticulum or Golgi apparatus, potentially involving unidentified signaling pathways. However, additional experimental validation is necessary to clarify the specific organelles and mechanisms involved in NINJ1 expression.

## Data Availability

Not applicable.
